# Velvet antler polypeptide combined with calcium phosphate coating to protect peripheral nerve cells from oxidative stress

**DOI:** 10.1007/s10735-022-10099-1

**Published:** 2022-08-29

**Authors:** Renqun Mao, Dalian Du, Xiaodi Zhu, Wenqing Li

**Affiliations:** 1 Department of Hand-Foot Microsurgery, Huazhong University of Science and Technology Union Shenzhen Hospital, Shenzhen, 518052 People’s Republic of China; 2 Department of Gynaecology, Shenzhen Nanshan District Maternal and Chlid Health Care Hospital, Shenzhen, 518000 People’s Republic of China

**Keywords:** CaP, RSC96 cell, Velvet Antler Peptide, Adhesion, Oxidative stress, Proliferation

## Abstract

Functionalizing biomaterial substrates with biological signals shows promise in regulating cell behaviors through mimicking cellular microenvironment. Calcium phosphate (CaP) coating is an excellent carrier for immobilizing biological molecules due to its non-toxicity, good biocompatibility, biodegradability, and favorable affinity to plenty of molecules. In this study, we reported the adhesion, the viability and proliferation behaviors after oxidative stress injury of Schwann cells RSC96 on CaP immobilized with the Velvet Antler Peptide (VAP) isolated from velvet antler through coprecipitation process in modified Dulbecco’s phosphate-buffered saline (DPBS) containing VAP. This approach provided well retention of functional molecules up to 28 days, and supported the adhesion and proliferation of RSC96 after oxidative stress injury without cytotoxicity. The simple and reproducible method of coprecipitation suggests that CaP is an ideal carrier to functionalize materials with biological molecules for peripheral nerve repair-related applications.

## Introduction

Peripheral nerve injury (PNI) is one of the most common, frequently occurring and difficult diseases in surgical clinics (Wang et al. 2019). Once the peripheral nerve is injured, the clinical repair effect is not satisfactory, which often results in patients with motor or sensory dysfunction or even disability, which brings life and health problems to patients, and also causes huge social pressure and economic burden. The difference between the peripheral nerve and the central nervous system is that the peripheral nerve can regenerate after injury. After peripheral nerve injury, Wallerian degeneration (WD) occurs at the distal end, which is considered to be an important step in the regeneration of damaged nerves. Wallerian degeneration takes precedence over nerve regeneration (Shen et al. 2018). During this process, Schwann cells (SCs) can help macrophages to engulf myelin fragments through autophagy, thereby accelerating the process of nerve recovery. This shows that the survival of SCs after an injury is also indirectly affected nerve repair and regeneration (Chen et al. 2017).

Due to the rapid growth of velvet antlers, it has attracted the attention of many researchers. Studies have shown that velvet antler is rich in amino acids, the content is as high as about 50%, there are at least 17 types, and it contains protein, phospholipids, cholesterol, sphingomyelin, and ganglioside, ceramides, polyamines, chondroitin sulfate A, estrone, estradiol, prostaglandins PGE1, PGE2, etc. and 20 kinds of trace elements such as calcium, phosphorus, magnesium, and copper (Tseng et al. 2016). Velvet Antler Peptide (VAP) isolated from velvet antler. It is composed of a variety of amino acids and is soluble in water. After research, VAP can significantly accelerate the regeneration of peripheral nerve tissue and bone tissue and wound healing, and has the effects of promoting the proliferation of epidermal cells, chondrocytes, and fibroblasts, as well as the differentiation of bone marrow mesenchymal stem cells (Huo et al. 2014; Zhao et al. 2016). Polylactide-glycolide microspheres equipped with VAP, combined with bone marrow mesenchymal stem cell transplantation, can significantly reduce the expression level of endoplasmic reticulum stress marker proteins, and inhibit the stress state to a certain extent, thereby repairing Sciatic nerve injury (Shao et al. 2012; Chen et al. 2009).

The growth of nerve cells plays an important role in nerve repair (Qin et al. 2016). The use of materials with good biocompatibility and biodegradability, and the modification of specific biomolecules on the surface of the material, thereby creating a physiological environment for the growth of nerve cells on the surface of the material, becoming an important means of regulating nerve cell behavior and promoting nerve repair (Ishimaru et al. 2018). At present, the commonly used methods for immobilizing biomolecules on the surface of biological materials are mainly physical adsorption, chemical grafting and carrier system methods. The carrier system method can significantly improve the low physical adsorption rate, easy diffusion and cumbersome chemical grafting when immobilizing biomolecules (Igberase et al. 2017). The limitations of this technology have become an important strategy for the development of simple and effective immobilization. Calcium phosphate (CaP) is a naturally occurring inorganic material that has excellent biocompatibility and good biodegradability and has an affinity for a large number of molecules (Eliaz and Metoki, 2017). It is widely used in the research of biomedical carrier systems (O’Neill et al. 2017). Therefore, the calcium phosphate coating combined with velvet antler polypeptide. It may have good application prospects in regulating the behavior of SCs.

In this study, the biomimetic mineralization method was used to load the velvet antler polypeptide on the calcium phosphate coating on the titanium surface to construct the velvet antler peptide/calcium phosphate functional coating. This functionalize coating not only effectively and continuously relases VAP, but also facilltates the adhesion, growth and proliferation of RSC96 cells and demonstrated great prospects in the field of sustained release of functional drug molecules, wound healing, tissue engineering, and peripheral nerve repair.

## Materials and methods

### Materials

Dulbecco’s phosphate-buffered saline (DPBS, without calcium and magnesium) was purchased from Gibco (Paisley, PA4, UK). CaCl_2_ solution, NaOH beads, acetone and absolute ethanol were obtained from Sigma-Aldrich (St. Louis, MO, USA). VAP and VAP-FITC were synthesized by Mingze Biotechnology (Xian, China). Micro BCA protein assay kit (mBCA), rhodamine-conjugated phalloidin and live/dead viability/cytotoxicity kit were obtained from Thermo Scientific (Rockford, IL, USA). RSC96 cells were obtained from Cell Bank of the Chinese Academy of Sciences (Shanghai, China). Phosphate-Buffered Saline (PBS), Penicillin–Streptomycin (Pen-Strep), Fetal Bovine Serum (FBS) and Dulbecco’s Modified Eagle’s medium (DMEM) were purchased from Hyclone (Logan, UT, USA). 0.25% trypsin–EDTA, 4% paraformaldehyde solution, Triton X-100, nuclei visualization solution DAPI (6-diamidino-2-phenylindole), Cell Counting Kit-8 (CCK-8) and reactive oxygen species assay kit were obtained from Beyotime Biotechnology (Shanghai, China). All cell culture dishes and multi-well plates were purchased from Corning (NY, USA).

### Preparation of calcium phosphate coating and loading VAP

Titanium substrates coated with calcium phosphate (CaP) and loading VAP were prepared by alkali treatment and biomimetic precipitation. CaCl_2_ solution (100 mg/L) was added in DPBS to prepare the modified DPBS (mDPBS) solution. Stock VAP solution (5 mg/mL) was diluted to 200, 400, 600, 800 µg/mL in mDBPS, and these diluted solutions were denoted as VAP200, VAP400, VAP600, and VAP800. The titanium substrates were first activated as below. Commercially pure titanium discs (grade IV, 10 mm in diameter, 2 mm in thickness) were ultrasonically washed in acetone, absolute ethanol, distilled water for 15 min each, and then soaked in 5 mL of 1 M NaOH solution at 140 °C for 6 h. For CaP coating precipitation, the activated titanium was immersed in 5 mL of mDPBS at 37 °C for 24 h. For further loading of VAP, the bionic calcium phosphate coated titanium was immersed in 1 mL of diluted VAP solution at 25 °C for another 24 h. The surface morphologies were taken from random fields of CaP, VAP200, VAP400, VAP600, and VAP800 by scanning electron microscope (SEM, Quanta 200; FEI Company, Philips, Netherlands).

### Amount of loaded VAP

The amount of loaded VAP was quantified by subtracting the amount of residual VAP from that of the initially added VAP using mBCA assay. Absorbance was measured at 562 nm using an ultraviolet/visible spectrophotometer (Shimadzu, Kyoto, Japan) and then was converted to VAP concentration using an albumin standard curve. The surfaces of CaP, VAP200, VAP400, VAP600, and VAP800 were then evaluated by Energy dispersive spectrum and X-ray photoelectron spectroscope (XPS, PHI-5300 ESCA) to study the chemical composition. The data were acquired by using A1 Kα X-rays with the photoelectron take-off angle being set at 45°.

### In vitro release of loaded VAP

To quantify the release kinetics of coprecipitated VAP from CaP, we chose FITC-VAP as the FITC tag allowed VAP amount to be measured through fluorescent spectrophotometry. Titanium substrates coated with CaP/VAP600 were incubated in 1 mL of PBS at 37 °C for 28 days under gentle orbital shaking (HNY-100D, Tianjin Honour Instrument Co., Ltd, China). At specific time points, the supernatant was completely collected and fresh PBS was replenished. The amount of released FITC-VAP was measured at Ex/Em = 495 nm/519 nm by microplate reader (VL0000D0, Thermo Scientific, USA).

### Cell adhesion

Schwann cells RSC96 were cultured in DMEM supplemented with 10% FBS and 1% Pen-Strep antibiotic antimycotic solution incubated in 37 °C, humidified, 5% CO_2_ environment. Cells of passage 3 ~ 7 were used for further cell experiments. Each CaP coated and VAP loaded titanium disc (CaP, VAP200, VAP400, VAP600 and VAP800) was sterilized in 75% ethanol, washed with distilled water, and placed under ultraviolet light overnight. Cells were seeded on samples at a cell density of 4 × 10^5^ cells/mL. Cells cultured on tissue-culture treated polystyrene (TCP) surfaces were used as a control (CTR). For adhesion assay, initial cell adhesion was measured for 4-h cultivation. After gently washing samples with PBS three times to remove non-adhered cells, the attached cells were measured by CCK-8. Brifly, after 4 h of incubation, 250 µL of fresh growth medium with 25 µL of CCK-8 reagents were added to each sample. The cell culture plates were incubated under the same cultivation conditions for another hour, and then reagents were carefully transferred to 96-well plates. The absorbance was measured using a microplate reader (ELx808; BioTek Instrusments, USA) at 450 nm. To observe the morphology of adherent cells on the samples, cells were fixed with 4% paraformaldehyde solution. The cells were then permeabilized using 0.5% Triton X-100. Subsequently, the cytoskeleton was stained by rhodamine-conjugated phalloidin for 30 min followed by counterstaining with 4′,6-diamidino-2-phenylindole (DAPI) to visualize the nuclei. Finally, the fluorescent images were taken through a laser scanning confocal microscope (LSCM, Nikon A1, Japan) in random field.

### Cell cultivation under oxidative stress

Samples (CaP, VAP200, VAP400, VAP600 and VAP800) were sterilized as the above described. RSC96 cells were seeded on samples at a cell density of 4 × 10^5^ cells/mL. Cells cultured on TCP surfaces were used as a negative control (CTR-). Cells cultured on TCP surfaces and then co-cultured with 10 mM H_2_O_2_ for 8 h were used as a positive control (CTR +) (Liu et al. 2017). Cells viability was assessed using CCK-8 and the live/dead viability/cytotoxicity kit, where RSC96 cells were cultured on samples for 24 h and then co-cultured with 10 mM H_2_O_2_ for 8 h. For the quantitative assay, NSCs viability was evaluated by CCK-8 as described above. For the qualitative assay, cytotoxicity was analyzed using the live/dead viability/cytotoxicity kit, according to the manufacture’s protocol (Sena-Lopes et al. 2020). Briefly, cells were washed with PBS followed by the addition of 2 µM Calcein AM and 4 µM ethidium homodimer (EthD-1). After incubation of 30 min at 37 °C, the cells were observed under a confocal microscope (Nikon A1, Japan), with excitation and emission of green (ex/em 494/530 nm for Calcein AM) and red (ex/em 528/645 nm for EthD-1) fluorescence. On the other hand, reactive oxygen species assay kit was used for ROS detection. Briefly, cells were washed with PBS followed by the addition of DCFH-DA probe diluted in 1:1000. After incubation of 30 min at 37 °C, the cells were observed under a laser scanning confocal microscope (LSCM, Nikon A1, Japan), with excitation and emission of green (ex/em 494/530 nm) fluorescence.

### Cell proliferation under oxidative stress

Longer-term proliferation was investigated by allowing cells to grow on samples for 1, 3, 5, 7 days after 10 mM H_2_O_2_ treatment as described above, with changing medium every 3 days. At predetermined time point, samples were assessed by CCK-8.

### Statistical analysis

All quantitative data were obtained from two or more independent experiments with triplicate or quadrant repeats and expressed as the mean ± standard deviation. Tests of significance were performed using Student’s t-test.

## Results and discussion

### SEM and chemical composition characterization

**Fig. 1:**
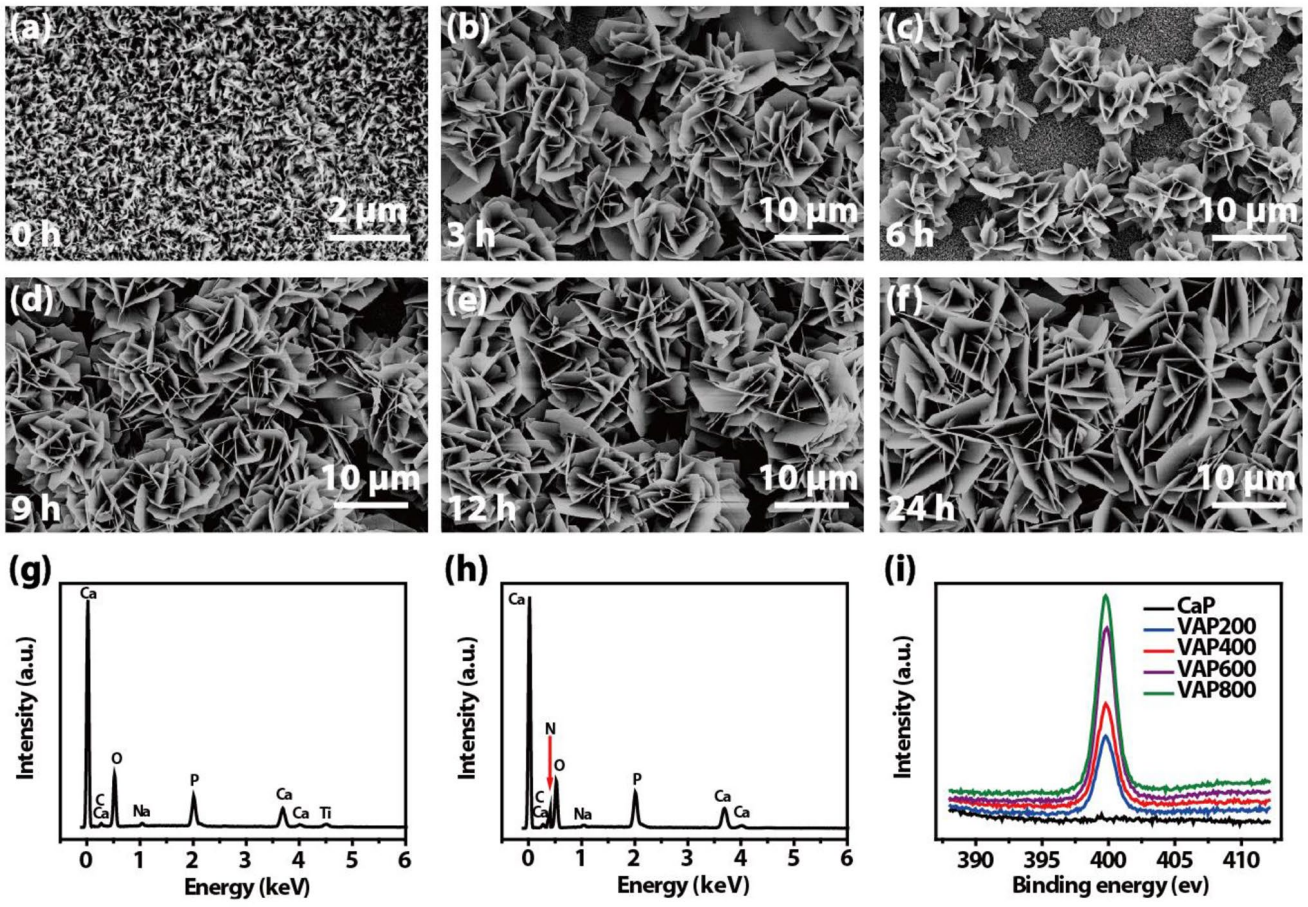
**a**–**f** SEM images of surfaces morphologies of CaP/VAP coating with various reaction time. EDS analysis of (**g**) CaP coating and (**h**) CaP/VAP (VAP600), (**i**) XPS analysis of N peaks in the CaP coating and CaP/VAP (VAP200, VAP400, VAP600, VAP 800)

The biomimetic mineralization method combining thermo alkali treatment and biomimetic precipitation was used to prepare functional coatings loaded with CaP/VAP on the surface of titanium (Nishiguchi et al. 2002; Schwarzkopf et al. 2011; Yu et al. 2012). In the process of bionic mineralization, as the reaction time increases, a layer of white coating visible to the naked eye gradually appears on the surface. Thermo alkali treatment not only corroded the oxide layer to obtain many submicron pore and loose structure, but caused the precipitation of titanium ions, resulting in the surface transformation into titanate-rich gel structure, which provided an abundance of crystal nucleation sites for calcium phosphate (Fig. 1a). During this hydration reaction, the anions such as TiO_4_^4−^, HTiO^3−^ and TiO_3_^2−^ generated on the surface are easily combined with Na^+^ to form titanate, after drying and losing water, these titanates were deposited on the surface of activated titanium sheets in the form of sodium titanate.

Figure 1b–f exhibits the micromorphologies of the functional coating on the surface of titanium disc with different coating time (3 h, 6 h, 9 h, 12 h, 24 h). After immersing in mDPBS solution for 3 h, lamella structure crystals can be observed, which sparsely cover the surface of the titanium sheet. As the reaction time increases, the length of the lamella structure gradually increases and cover the surface. After 12 h of reaction, the lamella structure of the titanium surface is relatively compact, and its size reaches the peak after 24 h. The formation of CaP coating on the surface of titanium sheet is related to the interaction of ions in the solution with its surface.

During the biomimetic precipitation, the anions such as TiO_4_^4−^, HTiO^3−^ and TiO_3_^2−^ near the surface of the titanium sheet attract each other, causing Ca^2+^ to gradually accumulate near the surface of the titanium sheet, partially forming calcium titanate and making the surface of the titanium sheet gradually positive potential, thereby attracting anions such as HPO_4_^2−^ and PO_4_^3−^ in the mDPBS solution under electrostatic force; at the same time, due to the local Ca^2+^ concentration is too high, Ca^2+^ combines with HPO_4_^2−^ and PO_4_^3−^ and other anions. This process was repeated, and then deposits on the surface of the titanium sheet to become the CaP crystal growth site (Ban and Hasegawa, 2002; Liu et al. 2002). In addition, the VAP present in the mineralized solution was involved in the entire biomimetic precipitation process, which is well loaded by the combination of physical adsorption and the carrier system to obtain the CaP/VAP coating.

The EDS analysis of CaP coating and CaP/VAP coating is shown in Fig. 1g, h. Ca, P, C, O, Na, and Ti elements were detected in the CaP coating, and Ca, P, C, O, Na, and N elements were detected in the CaP/VAP coating. EDS has limited detection of coating thickness. CaP/VAP coating maybe a little thicker than pure CaP coating, so the substrate Ti element cannot be detected. At the same time, EDS is difficult to detect light elements such as H and N, and because the protein content that can be loaded in the CaP/VAP coating is less, the detection intensity of N element in Fig. 1h is low. To a certain extent, this shows that the VAP molecules are successfully loaded in the coating.

The XPS spectra in Fig. 1i revealed the change in surface chemical compositions caused by coprecipitation of biological molecules. The N 1 s peaks (398.69 eV) originated from nitrogen components of VAP were detected in the XPS spectrum from CaP with coprecipitated VAP (VAP200, VAP400, VAP600 and VAP800). The magnified XPS spectrum of N peaks displayed that nitrogen intensity increased with increasing initial VAP concentration in mDPBS solution. During the whole experiment, only VAP molecule contained N element, so we once again proved that VAP was successfully loaded into the coating by XPS detection.

### Quantification and release kinetics of loaded VAP

**Fig. 2:**
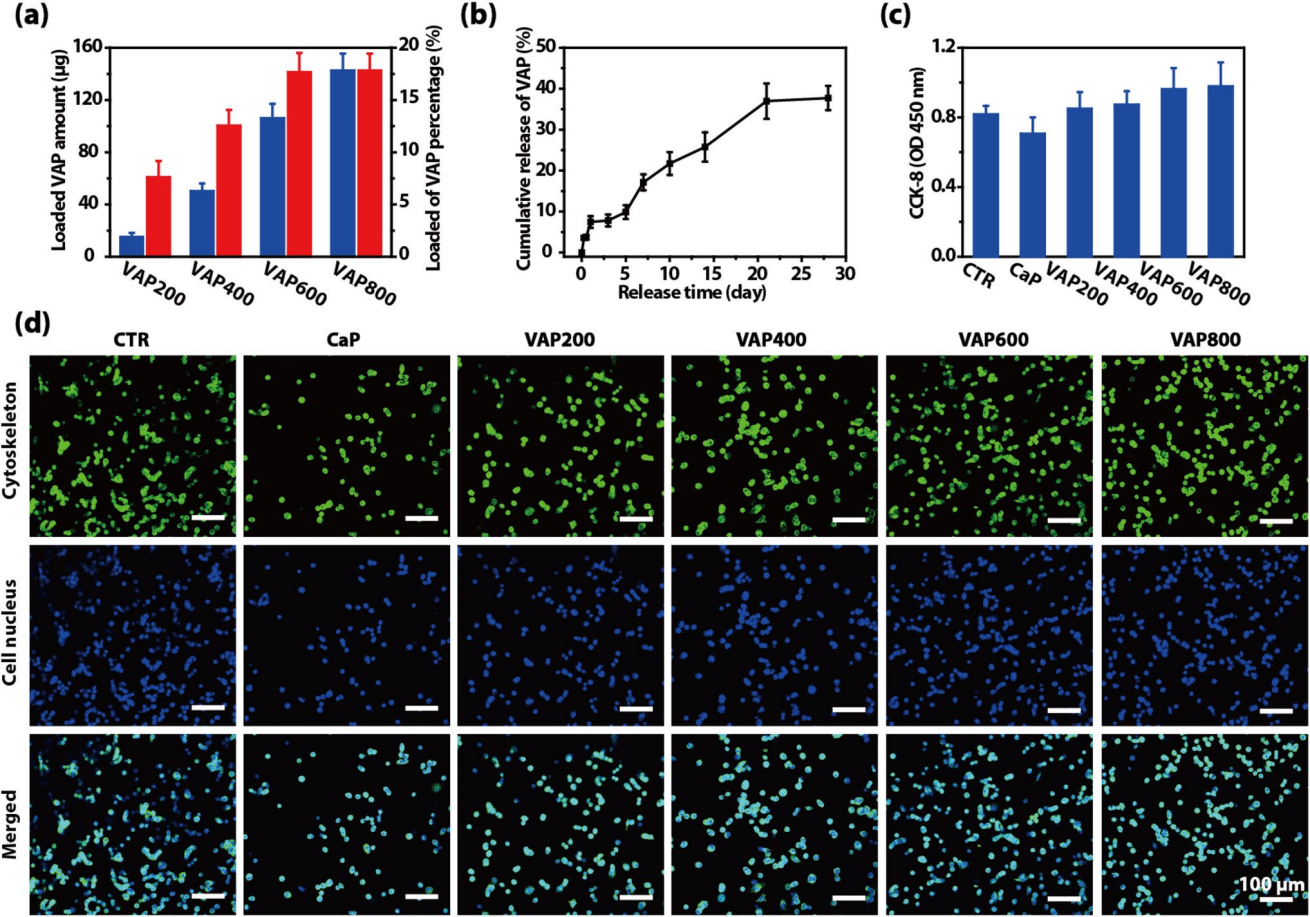
**a** Amounts of VAP in various CaP/VAP. **b** in vitro release of FITC-VAP in CaP/VAP600 in PBS over 28-day time period. **c** Quantitative datas and **d** LSCM images of RSC96 cell adhesion on surfaces of different samples after 4 h culture

Quantification of loaded VAP by the mBCA assay, that is, based on the concentration difference between the inital concentration of VAP in mDPBS solution and after immersing CaP coated titanium for 24 h. Figure 2a shows the amount of coprecipitated VAP on each sample with the different initial concentrations of VAP. The amount of coprecipitated VAP increased from approximately 15.19 μg (7.59%) to nearly 142.64 μg (17.83%), with increasing initial concentration of VAP from 200 to 800 μg/mL. The experimental design and XPS data were verified by quantitative test based on mBCA assay.

The retention of the immobilized protein is critical for practical application. We investigated the release kinetics and retention of coprecipitated FITC-VAP in a biological environment. Titanium substrates coated with CaP/VAP600 were immersed in PBS solution for 28 days. The release kinetics of VAP from CaP is depicted in Fig. 2b. The FITC-VAP was released stably and constantly. After 6 and 12 h, 3.72 ± 0.40 and 3.79 ± 0.58% of coprecipitated FITC-VAP were released into PBS solution, respectively. Even after 28 days, only approximately 37.71% of FITC-VAP immobilized within CaP was released into the solution. It has been confirmed that the degradation of CaP in neutral solution (Su et al. 2013), together with diffusion of molecules from biomimetic CaP can successfully control the slow release of VAP for long term (28 days). This indicated that coprecipitation of biological molecules within biomimetic CaP may provide spatial control of cell behaviors through a stable and constant dosage of immobilized molecules.

### Initial cell adhesion

The initial cell adhesion is a crucial early step in the migration and proliferation in tissue repair in vivo (Iijima et al. 2017; Wong et al. 1985). The CaP/VAP at various amounts (CaP, VAP200, VAP400, VAP600 and VAP800) were assayed for RSC96 cells adhesion. Cells were seeded on b CaP/VAP in a growth medium for a period of 4 h, and the results of cell adhesion are summarized in Fig. 2c, d. Compared with CTR, VAP200, VAP400, VAP600 and VAP800 samples show comparable ability to promote RSC96 cells attachment. Compared with CaP, the achieved amounts of loaded VAP were high enough to improve RSC96 cell adhesion. This may be since VAP is a good bioactive substance and part of VAP is released from CaP coating within 4 h. The results of cytoskeleton staining by rhodamine-conjugated phalloidin and DAPI staining after 4 h of cell growth (Fig. 2d) showed that the number of cells on the CaP/VAP coating (VAP200, VAP400, VAP600 and VAP800) was significantly higher than that on the pure CaP coating, which was consistent with the detection results of CCK-8 assay. Moreover, the cells attached to the CaP/VAP coating spread well, comparable to the CTR. These results indicate that the continuous release of VAP molecules by the CaP/VAP coating can form an environment that promotes the growth of nerve cells, which indicates that the CaP/VAP coating loaded with VAP is a suitable carrier system for the adhesion and growth of nerve cells, and has certain neuro compatibility.

### Cell cultivation under oxidative stress

Previous studies have shown that VAP can significantly reduce the expression level of endoplasmic reticulum stress protein and inhibit its stress state to a certain extent, to repair sciatic nerve injury (Zhang et al. 2017). We further investigated the RSC96 cell viability of growth on CaP/VAP coating (CaP, VAP200, VAP400, VAP600 and VAP800) by means of the live/dead viability/cytotoxicity kit and CCK-8.

**Fig. 3:**
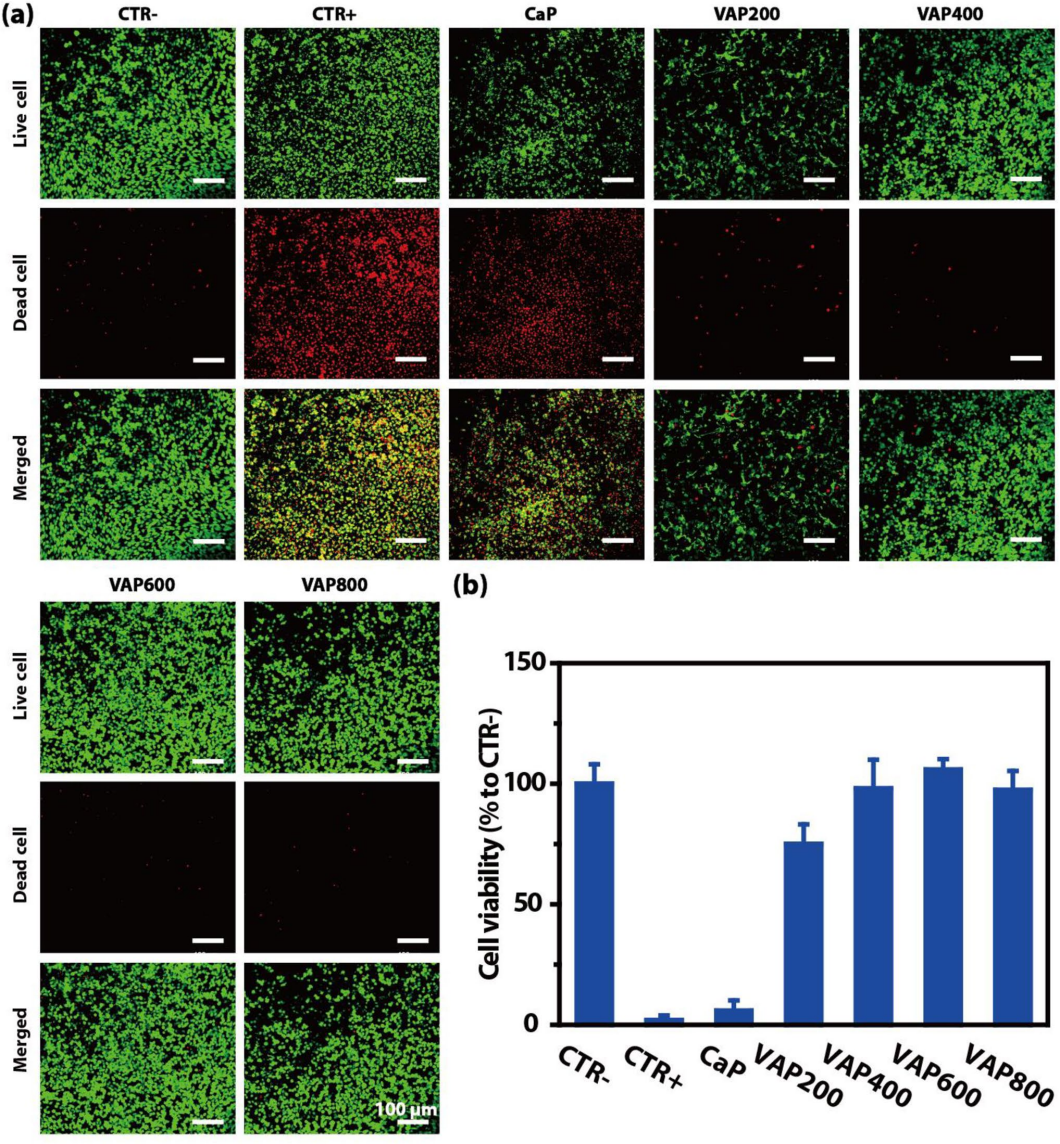
**a** LSCM images and **b** quantitative datas of RSC96 cell viability on surfaces of different samples after 24 h and then co-cultured with 10 mM H_2_O_2_ for 8 h. culture

Figure 3a shows live (green)/dead (red) stain of RSC96 cell under oxidative stress cultivation. The live/dead viability/cytotoxicity kit provides a two-color fluorescence in cell viability test. Live cells with intracellular esterase activity could converse the nonfluorescent cell-permeant calcein AM to fluorescent calcein, which show green fluorescence (Zhou et al. 2011). On the other hand, EthD-1 enters dead cells with damaged membranes and undergoes enhancement of fluorescence with binding to nucleic acids, thereby producing red fluorescence in dead cells (Gantenbein-Ritter et al. 2011). For VAP200, VAP400, VAP600 and VAP800, green fluorescence emission signal was dominated and comparable with CTR-. But for CTR + and CaP, red fluorescence emission signal was dominated and comparable with CTR + .

As shown in Fig. 3b, after 10 mM H_2_O_2_ treatment for 8 h, CCK-8 assay of RSC96 cell revealed that cell viability compared with CTR-, cells cultured on TCP surfaces without H_2_O_2_ treatment, was 75.10%, 98.02%, 105.90% and 97.38% for VAP200, VAP400, VAP600 and VAP800, respectively. For CTR + and CaP, cells cultured on TCP surfaces or CaP and then co-cultured with 10 mM H_2_O_2_ for 8 h, cell viability was decreased to 2.02% and 5.90%, respectively. The results were consistent with those of the live/dead viability/cytotoxicity kit.

These results indicate that VAP within CaP coating continuously released within 24 h has a protective effect on RSC96 cells, which can maintain the normal viability of the cells when they are damaged by oxidative stress in the later stage.

Reactive oxygen species (ROS) include singlet oxygen, hydroxyl free radical, hydrogen peroxide, nitric oxide, superoxide anion, peroxide hydroxyl free radical, peroxide-free radical and so on. These ROS affect every process of cells from growth and development to proliferation, aging and even death (Ray et al. 2012). We used DCFH-DA probe to detect intracellular ROS. DCFH-DA itself does not have fluorescence, but it can pass through the cell membrane freely. After entering the cell, enzymes in the cell hydrolyze it to DCFH, and then it can be oxidized by intracellular reactive oxygen species to become DCF with fluorescence characteristics. The probe emits green fluorescence, the intensity of which is positively correlated with the level of active oxygen in the cell (Huang et al. 2016).

**Fig. 4:**
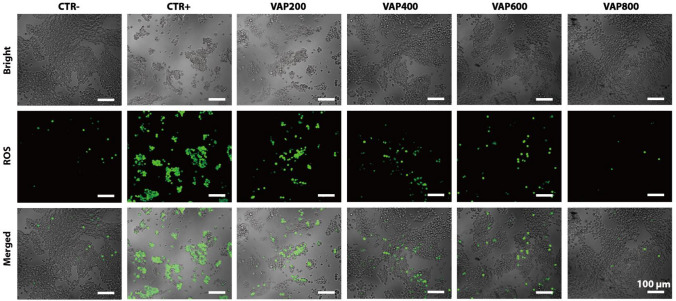
LSCM images of RSC96 cell ROS measurement on surfaces of different samples after 24 h and then co-cultured with 10 mM H_2_O_2_ for 8 h culture

As shown in Fig. 4, ROS staining results show that RSC96 cells growth with VAP200, VAP400, VAP600 and VAP800, the production of ROS decreased gradually. The cell morphology of the VAP600 and VAP800 samples was similar to that of the CTR-, with very few ROS produced. This further indicates that the slow release of VAP can protect RSC96 cells from oxidative stress, and is positively correlated with the release amount of VAP.

### Cell proliferation under oxidative stress

**Fig. 5:**
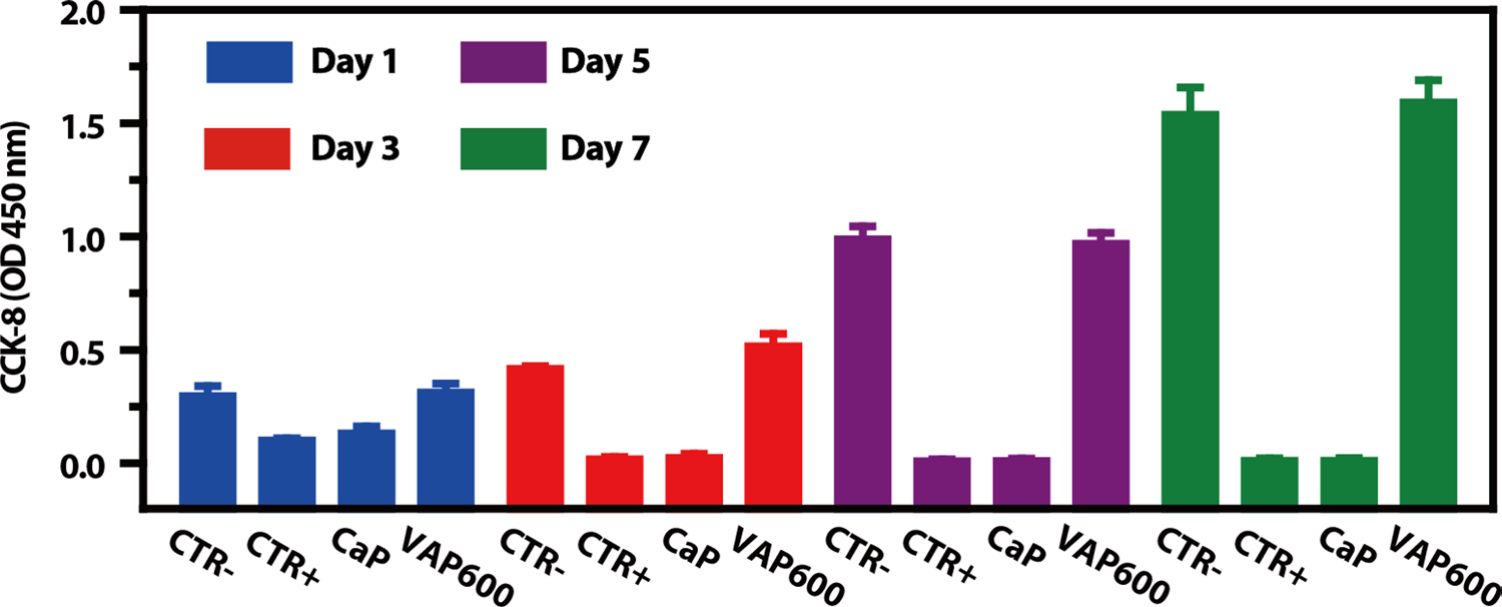
RSC96 cell proliferation under oxidative stress for 1, 3, 5, 7 days

In order to determine whether VAP coprecipitated CaP can support long-term RSC96 cells culture after oxidative stress injury, the proliferation of cells cultivated on CaP and VAP600 over 7 days was investigated. At the predetermined time point, the cell viability was evaluated by CCK-8 (Fig. 5). The cells proliferated well on VAP600 coating for 1, 3, 5 and 7 d, as the CTR-. The cells growth on CaP coating showed no proliferated, due to the CaP coating without VAP, could not protect cells from oxidative stress and maintain the normal viability, as the CTR + . These results indicate that CaP/VAP coating can maintain the growth and proliferation of nerve cells RSC96 cells after oxidative stress injury, while CaP/VAP coating loaded with VAP shows a good ability to promote the proliferation of nerve cells RSC96 due to the slow and continuous release of VAP molecules on its surface.

To protect peripheral nerve cells from oxidative stress, a surface modification which provides stable cell-surface adhesion and promotes cell proliferation is crucial (Li et al. 2017). Using biocompatible and biodegradable materials and modifying specific biomolecules on the surface of the materials to create a physiological environment for the growth of nerve cells has become an important means to regulate the behavior of nerve cells and promote nerve repair. Studies have shown that VAP can significantly accelerate the repair of peripheral nerve tissue after injury.

## Conclusion

In summary, we have successfully synthesized a the velvet antler peptide/calcium phosphate functional coating by the biomimetic mineralization method. The present study illustrates that the CaP with loaded VAP is neither cytotoxic nor has negative influences on RSC96 cell adhesion. Moreover, the CaP with loaded VAP can effectively maintain cell viability and normal proliferation and growth for a long time after oxidative stress injury. Therefore, loading biological molecules (VAP) within CaP through the simple and reproducible biomimetic mineralization method is promising in the field of sustained release of functional drug molecules, wound healing, tissue engineering, and peripheral nerve repair.

## Data Availability

The datasets used and/or analyzed during the current study are available from the corresponding author on reasonable request.
